# Editorial: Neurosurgery and Neuroanatomy

**DOI:** 10.3390/brainsci12030341

**Published:** 2022-03-02

**Authors:** Kaan Yağmurlu

**Affiliations:** Department of Neurological Surgery and Neuroscience, University of Virginia School of Medicine, Charlottesville, VA 22903, USA; ky7zb@virginia.edu

Microsurgical anatomy is not only the backbone for neurosurgical operations, but also for technological innovations, novel surgical techniques, a better understanding of the etiopathogenesis of pathologies, and translational medicine from neuroscience to daily clinical practice.

The overarching goal of this Special Issue was to build a bridge between research and patient care studies and to bring neuroscientists and clinicians together.

This Special Issue contains 16 individual papers that are categorized into research laboratory investigations; clinical case series, including those of the brain and spine regarding updated intraoperative technologies; novel neurosurgical tenets; and molecular anatomy.

Wysiadecki et al. [1] contributed two papers about skull base surgery. Their contribution “Gross and Micro-Anatomical Study of the Cavernous Segment of the Abducens Nerve and Its Relationships to Internal Carotid Plexus: Application to Skull Base Surgery” is a microsurgical and histological study of the cavernous segment of the abducens nerve that is considered a blind spot and that is vulnerable during endoscopic or transcranial skull base surgery. This study enlightens the course of abducens nerve course and subdivision to avoid iatrogenic injury. The second article [2] by the same group titled “Microsurgical Anatomy of the Inferomedial Paraclival Triangle: Contents, Topographical Relationships and Anatomical Variations” is about the microsurgical and histological examination of the structures forming the inferomedial triangle of the petroclival region via quantitative measurements. Petroclival tumors are challenging to remove, and it is essential to be aware of the relevant structures mentioned in this article in order to avoid complications. 

The article published by Poblete et al. [3], simplifies the complexity of anterior vascular circulation by correlating microsurgical anatomy with neuroimages to provide a better understanding on this phenomenon.

Anterior petrosectomy provides excellent exposure to the petroclival region, but its related morbidity is non-negligible. The paper published by Flores-Justa et al. [4] notes safe practices for anterior petrosectomy (Kawase’s approach) with the aid of a neuronavigation device for the petroclival region. They demonstrate the surgical procedure step by step with excellent dissection pictures that correlate with CT neuronavigation pictures. This study highlights the pertinent neurocritical structures and describes how to avoid harming them.

The article published by Çevik et al. [5] examines the anatomic variations in the cranial nerves that are encountered during carotid endarterectomy, such as the hypoglossal, vagus, and mandibular branch of the facial nerves, in twenty-two cadaveric human heads. It is crucial to be aware of the variations noted in this study to avoid injuring the cranial nerves.

Göksu et al. [6] published the clinical outcomes of endoscopic optic nerve decompression using their opening nerve sheath technique in patients with idiopathic intracranial hypertension, which resulted in an improvement in visual acuity (78%), visual field defect (62.5%), and papilledema (100%). Their surgical technique was demonstrated using cadaveric dissection and intraoperative video.

The paper published by Kim et al. [7] is a clinical series discussing the placement of a silicone elastomer sheet in the epidural space during decompressive surgery to diminish the intra- and postoperative complications caused by epidural adhesion in patients undergoing cranioplasty surgery. They statistically compared two patient groups, one using the silicone elastomer sheet and one control group, and concluded that utilizing the sheet facilitated cranioplasty surgery by shortening the operation time, reducing the estimated blood loss, and minimizing the long-term complication of epidural fluid collection that is common during this type of operation.

Revascularization procedures are considered to be the most effective treatment modality that can be implemented to diminish the risk of intracranial hemorrhages in hemorrhagic moyamoya disease. On the other hand, delayed anastomotic occlusion is a common long-term complication after direct vascularization procedures. In the article published by Chen et al. [8], the authors statistically evaluate the clinical experience of 87 adult patients with moyamoya disease. They conclude that delayed anastomotic occlusion has no significant correlations with long-term angiographic and neurological outcomes, except neoangiogenesis.

Extreme lateral interbody fusion (XLIF) has become a standard technique for the fusion of the thoracic and lumbar spine [9]. It is an effective technique that allows indirect nerve decompression and the restoration of the spinal foramen and the disc space height by routing through the psoas muscle. The proximity of the psoas muscle with the lumbar plexus might cause postoperative neuropathic pain such as anterior thigh pain; sensory disturbances; and muscle weakness in hip flexors. In the article published by Yingsakmongkol et al. retrospectively evaluated psoas muscle volume as a possible predictive factor for the occurrence of anterior thigh symptoms and concluded that there no relationship could be observed between them in 81 consecutive patients.

The article published by Pojskić et al. [10] is a prospective study focusing on the use of intraoperative computer tomography-guided augmented reality, which provides a better understanding of 3D anatomy to avoid severe complications, for lateral interbody fusion cases with various indications, including oncological and infectious spinal diseases.

The paper “Do Orthopedic Surgeons or Neurosurgeons Detect More Hip Disorders in Patients with Hip-Spine Syndrome? A Nationwide Database Study” by Yin et al. [11] pointed out the differences between the neurosurgeons and orthopedic surgeons in terms of the rates at which they diagnose hip disorders based on a retrospective study with 1824 patients. Since the degenerative hip and spine pathologies can mimic each other both in terms of history and physical examination misdiagnoses and wrong surgical decisions with unsatisfied surgical outcomes are possible. Based on patient history and physical examinations, the authors highlight the importance of correct radiographic studies to examine both lumbar spine and hip pathologies.

Soldozy et al. [12] systemically reviewed the current state of the literature focusing on the diagnostic, surgical, and technical considerations for lumbar interbody fusion in patients with osteopenia and osteoporosis, which predisposes postoperative instrumentation failure and low surgical outcomes. They concluded that the literature remains sparse as to what approach should be employed in this patient population. The authors also suggested that patients should be preoperatively screened in Hounsfield units for better outcome prediction.

“Spontaneous Resolution of Late-Onset, Symptomatic Fluid Collection Localized in the Meningioma Resection Cavity: A Case Report and Suggestion of Possible Pathogenesis” is the contribution by Kim et al. [13]. The authors share their experiences to warn neurosurgeons against unnecessary reoperation in such a case.

Transcranial MR-guided focused ultrasound has been adopted as a noninvasive ablative procedure for the treatment of movement disorders and psychiatric disorders [14]. Additionally, many experimental studies have been carried out on this technology. In the article published by Giammalva et al. reviewed clinical and experimental studies on transcranial MR-guided focused ultrasound technology to provide future directions for this technique.

“Common Challenges and Solutions Associated with the Preparation of Silicone-Injected Human Head and Neck Vessels for Anatomical Study”, the article in this Special Issue published by Çırak et al. [15], reports a colored silicone injection and the preservation of the cadaveric heads in detail and the technical shortcomings of standard injection procedures to overcome common challenges based on their experiences working with 35 heads over eight years. The authors also highlight the importance of using well-silicone injected heads for research and educational purposes.

Since the beginning of this century, the portion of the central nervous system that is associated with lymphatics has gained popularity after the proven existence and functionality of the lymphatic structures for cleaning waste products from cerebrospinal fluid in animal studies [16]. Animal studies have demonstrated the connection between the subarachnoid space and deep cervical lymph nodes via dural lymphatic vessels. Our human cadaveric study revealed the lymphatic channels that pass through the jugular foramen that connect to the cervical lymphatic network using immunohistochemical staining and microsurgical dissection. It was hypothesized that the demonstrated lymphatic channels might represent a possible route for the spread of cancers to and from the central nervous system.
